# Expression of Concern: Protective effects of *Lactobacillus rhamnosus* GG against human rotavirus-induced diarrhoea in a neonatal mouse model

**DOI:** 10.1093/femspd/ftaf007

**Published:** 2025-08-29

This is an expression of concern regarding: Zhen Zhang, Yun Xiang, Na Li, Baoxiang Wang, Hongwu Ai, Xiaomei Wang, Laiqiang Huang, Yi Zheng, Protective effects of *Lactobacillus rhamnosus* GG against human rotavirus-induced diarrhoea in a neonatal mouse model, *Pathogens and Disease*, Volume 67, Issue 3, April 2013, Pages 184–191, https://doi.org/10.1111/2049-632X.12030

Concerns regarding similarities within Figure 5 were raised on Pub Peer [https://pubpeer.com/publications/6F612B61669B506E40774D0B0E993F#] and subsequently were flagged to the Editor-in-Chief in 2025.

The journal attempted to contact the corresponding author but was unable to locate any contact information and is investigating our concerns about Figure 5 in line with COPE guidance. The journal is publishing this Expression of Concern to notify readers of the concerns whilst the outcome of the investigation is pending and advises readers to examine the details of this study with particular care.

